# Supplementary material for: Nanoscale mechanism of UO2 formation through uranium reduction by magnetite
Source: Nat Commun. 2020 Aug 10;11:4001. doi: 10.1038/s41467-020-17795-0 (PMC7417540; doi:10.1038/s41467-020-17795-0)
Supplement: Supplementary file 1 — Supplementary Information [file 41467_2020_17795_MOESM1_ESM.pdf]

Supplementary Information

Nanoscale mechanism of UO_2 formation through uranium reduction by magnetite

Pan et al.

Supplementary Notes

Supplementary Note 1. Selected area electron diffraction (SAED) measurement. The reduction of uranium oxides results in phase transitions which can be determined indirectly from crystallographic analysis of the structures in TEM samples. Thus, we endeavored to identify the valence state of U-bearing nanoparticles using electron microscopy and SAED. We performed a fast Fourier transform (FFT) analysis of the HR-STEM images shown in Figures 3 to 5 in the main text. While HR-STEM approaches can directly map structures of phases on the atomic structure, it is not a rigorous crystallographic analysis and can result in the ambiguous or incorrect identification of phases. Robust crystallographic identification of phases can be implemented by either performing Multi-Slice simulations of HRSTEM imaging, or by taking systematic SAED measurements of nanometric regions of sample materials. We chose SAED to complement our FFT analysis of HR-STEM.

Definitive identification of uranium oxide phases requires both the proper indexation of the SAED data and the comparison of diffraction data to standards with known phases and valence states. We prepared TEM specimens from UO_2, U_3O_8, UO_3 materials to compare with the 72-hour samples from the same stock used in the EELS measurements and microscopy investigations. We used the same methods for preparation as for the samples used for EELS measurements. The collected solids were dispersed into 70% ethanol solution, sealed in a serum bottle and sonicated for 3 min anoxically. A drop of the sonicated suspension was then deposited onto an ultra-thin carbon grid (Electron Microscopy Sciences CF200-CU-UL; 200 μm square mesh; 3-4 nm carbon foil; copper grid; silicon free) and was immediately transferred into a vacuum desiccator for preservation before the measurement. The sample spent less than 15 mins under ambient conditions before being introduced into the vacuum of the TEM.

We conducted SAED measurements on a JEOL 2100 LaB_6 operating at 200 kV. As noted for the EELS measurements, the TEM samples can incur damage from the electron beam. We cooled the samples to ~108K in a Gatan 620 LN2 cooling stage to reduce the effects of radiolysis and degradation of the samples under the electron beam. Colella et al. (2005) observed that the uranium oxide phase could have reduced under the electron beam at these temperatures with extended exposure and total doses. We minimized the total exposure time to a few minutes and limited the beam current on the sample to ~1nA, thus reducing the total dose below that of EELS measurements, ~$1x10^{25}$ electrons/m^2. For the SAED measurements in a low dose configuration, we used spot size #5 and inserted a small 70 μm condenser aperture, which produced beam currents of ~1 nA in a spread beam having a diameter ~ 2 μm (Koehler, parallel illumination condition). Under such microscope conditions, the beam damage was minimized, and most samples were stable and did not change phase or evolve within the 1 to 2 min exposure period. However, we did note changes to the UO_3 standard samples, which appeared to be more beam sensitive than the other samples analysed by SAED. During the short exposure, diffraction spots disappeared or changed symmetry, indicating that the UO_3 samples may have transformed under the electron beam. Diffraction analysis confirmed that not all diffraction reflections could be ascribed to the crystal structure of UO_3-1540845.

Simulated diffraction patterns were generated using JEMS software[1] and compared to the experimental patterns. To improve the analysis and phase identification, we radially integrated the experimental SAED patterns and plotted their diffraction intensity distribution with reciprocal space using the PASAD plugin within in Gatan DigitalMicrograph® software[2]. The following PDF crystal files data were used to generate the simulated patterns and to index the experimental patterns: UO_2-1541665, U_3O_8-2310811 and UO_3-1540845 from the Crystallography Open Database. Supplementary Figure 7 shows a montage of SAED patterns, and the profiles of simulated SAED patterns generated from the PDF files were overlaid and compared to the experimental data. Due to the large crystal sizes, the experimental SAED data of the UO_2 standard sample have a spotted pattern rather than a powder ring pattern (Supplementary Figure 7 a,b). The comparison shows that both UO_2 standard and nanowires (72-hour) structure SAED fit with the UO_2-1541665 PDF file simulated pattern (Supplementary Figure 7 c,d). A good fit to the PDF file U_3O_8-2310811 was found for U_3O_8 standard (Supplementary Figure 7 e,f). As for the UO_3 standard, the SAED pattern

changed during measurement, and the sample evolved under the electron beam, potentially incurring damage and changing phase and valence state. Analysis of the SAED revealed that there are missing and shifted reflections for the UO_3 standard data (Supplementary Figure 7 g,h). The behavior of this sample under the low-dose conditions of SAED measurements was distinctly different from that of other standards and the 72-hour samples. Though SAED data of UO_3 are fraught with artifacts, we present them here to underscore the necessity for rigor when analyzing these materials in TEM that can potentially reduce under the high-energy electron irradiation.

The scattered line plot in Supplementary Figure 8 and indexation of the patterns clearly show that the electron diffraction pattern of the nanowires in 72-hour sample contains reflections that can only be indexed to the UO_2 phase, and the pattern matches well the UO_2 standards and the PDF file UO_2-1541665 data. We, therefore, conclude that the nanowires primarily contain reduced U(IV), which also corresponds well and validates our complementary FFT analysis of HR-STEM images and electron energy loss spectroscopy (EELS) measurements.

Supplementary Note 2. Description and motivation for the defined EELS measurement parameters. The experimental determination of uranium valence state in oxides complexes follows closely the methodologies implemented in previous studies[3,4]. We adapted those approaches in these experiments due to specific experimental challenges, e.g., radiation damage and the difficulties posed by spatially probing different nanostructures comprising mixtures of uranium IV, V, and VI valence states. In general, investigating uranium reduction reactions with electron microscopy and electron energy loss spectroscopy (EELS) is challenging since the electron beam irradiation at high doses can directly reduce uranium, and the limitations in the EELS spectrometer hardware confine our observations to a limited number of approaches. As such, we developed rigorous methods to avoid experimental artifacts from beam-induced effects and to provide statistically relevant investigations and precise determinations of the valence states through the use of well-characterized uranium oxide standards. In the following, we detail a step-by-step procedure for determining the valence states by calculating the branching ratio between the M_4 and M_5 edges of uranium and comparing these ratios with well-characterized uranium oxide standards.

The branching ratio is calculated by measuring the integrated counts under the M edges and taking the ratio of $M_5/(M_4+M_5)$. This calculation is performed through a series of operations on the raw spectrum data (Supplementary Figure 9a). First, we subtract the background using the fitting algorithms contained within the Gatan DigitalMicrograph® software using a selection window on the pre-edge (Supplementary Figure 9b). Then, we take a second derivative of the background-subtracted spectrum using routines contained within the Gatan DigitalMicrograph® software package that calculates an approximate second derivative (Supplementary Figure 9c). The parameters of the second derivative calculation averages over an interval defined as 'w+' units wide, which are ideally set to match the edge width, and subtracts half of the averages in the two adjacent "wings" of the edges, having a width defined as 'w-' in the software. The spectrum is then divided by the squared sum of 'w+' and 'w-' to yield an approximation of the second derivative with respect to energy. For spectra measured at a dispersion of 0.25 eV/per channel, we used window widths of 5 and 10 eV, respectively, for 'w+' and 'w-'. In the final step, we determined the integrated counts under the positive peaks of the M_4 and M_5 edges and extracted the integral counts. Using the extracted integral counts, we calculated the branching ratio, $M_5/(M_4+M_5)$.

We chose to acquire spectra at dispersions of 0.25 eV/per channel which provided sufficient energy resolution to produce well-defined M edges above background and enough range to measure the pre-edge and post-edge backgrounds for both M_4 and M_5 edges in a single-spectrum. Because the electron loss is higher than 2,000 eV, the drift tube and prism excitations of the spectrometer had to be adjusted to provide the appropriate range between 3,450-3,940 eV. Thus, dual EELS acquisition techniques could not be used in which both the low loss regime with the zero-loss peak and high low loss spectrum with the M edges can be acquired simultaneously. Dual EELS acquisition is useful for calibrating the absolute edge energy position and determining chemical shifts in the edge energies associated with changes in valence. Combined with the use of the monochromator in the Titan Themis, it is possible to measure valence states from chemical shifts precisely and also differentiate them spatially using scanning TEM based techniques. However, the chemical shifts associated with different valence states in uranium are below 1 eV, which cannot be measured with Dual EELS acquisition. Furthermore, the use of high dispersion to observe both edges does not permit sufficient energy resolution for accurately determining the valence states from these methodologies. For these reasons, we adapted the branching ratio measurement schemes of Colella et al. (2005) to locally identify the valence states of different nanostructures within the samples.

Colella et al. (2005) also reported on the challenges of measuring valence states of uranium due to electron beam damage that can reduce the uranium in oxides, giving an observable change in the branching ratio. An inherent consequence of measuring valence states from inelastically scattered electrons is that the sample must be ionized by the beam. Furthermore, to obtain appreciable signals at the high loss M edges of uranium (≥ 3,550 eV), we must irradiate the sample with high doses. There is, thus, a trade-off between having sufficient signal-to-noise (SNR) in the measured edges and limiting the amount of beam-induced effects. As the samples in our experiments are dispersed nanostructures, the minuscule sample volume also complicates the

EELS measurements, and as a consequence, we must increase the local dose on the sample in the EELS measurements by using electron beam currents of several nano-amperes to obtain well-distinguished edges above background. Also, to obtain well-resolved edges, we used exposure times of 5 s, summed 10 spectra together and acquired a high-quality (HQ) dark reference to improve SNR and energy resolution. In utilizing these acquisition parameters, the samples were exposed for several minutes at high beam currents. These high doses reduce the TEM samples at ambient temperatures. According to the findings of Colella et al. (2005), which were based on the analysis of uranium oxide standards, an increase in the branching ratio signifies the reduction of uranium. To minimize the beam-induced effects that chemically reduce the sample through a combination of heat and ionization damage, we cooled the sample to 108 K in a liquid nitrogen specimen cooling stage and operated the microscope at high tension of 300 kV in TEM mode.

We used diffraction-coupled EELS geometry with small camera lengths of 29 mm to collect a significant amount of the inelastic signal, improving the SNR, and reducing the exposure time and total dose. Diffraction-coupled EELS also allowed to spatially isolate different regions of the sample that contribute to the spectrum data, and thus, we could probe variation in valence states between the different nanostructures. Using the smallest selected area electron diffraction (SAED) aperture of 10 μm, we isolated areas with a diameter of ~200 nm which was sufficient, e.g., to separately probe the valence states of uranium on magnetite particles and the U-containing nanowires (Supplementary Figure 9d,e). Ideally, the illumination of the sample should be parallel for diffraction-coupled EELS. However, we used a slightly converged beam (~1 mrad) for two primary reasons: (1) regions with suitable thickness and particle density were limited in the TEM samples for which we wanted to restrict and control the amount of dose in the surrounding areas that were interrogated in subsequent measurements, and (2) we needed to increase the current in the spectrometer to 2 nA, requiring us to converge the beam. To provide statistically relevant measurements, we used a fixed C2 lens current and illumination area. Having a 2 nA current in 200 nm diameter area gives an approximate dose rate of ~$4x10^{23}$ electrons/m^2/s. We aimed to have the high current illumination of the sample and maintain the measurement time under 2 minutes. This long exposure time includes both the acquisition of spectra and the setup of the measurement. To reduce noise and improve SNR, we used 5 s exposure and summed 10 spectra together using the automated algorithm within Digital Micrograph, and the total acquisition of the spectra was roughly one minute. Thus, we limited measurements to a total dose of ~$5x10^{25}$ electrons/m^2. Though no prior studies have been reported on the critical dose and dose rate, the measured branching ratios in our study suggest that the irradiation conditions under the liquid nitrogen conditions used in our experiments were below the threshold to induce the reduction of uranium oxides.

To provide internally consistent data sets and to account for beam-induced effects, we performed “benchmark” measurements on uranium oxide standards having known valence states at the same microscope conditions and temperatures and under the same dose and dose rate conditions. We obtained the following standards for the three different valence states, uraninite for U(IV), $UMoO_5$ for U(V), and UO_3 for U(VI). We performed a minimum of 10 measurements per standard. We chose regions in the TEM samples that were comparably as thin as the nanowire samples such that background signals and EELS edge shapes were similar. Using Gatan DigitalMicrograph® software, we processed the spectra under the same routines and determined the branching ratio the median values from 0.6956, 0.6887 to 0.6640, for the U(IV), U(V), and U(VI) standards, respectively (Supplementary Table 3). As discussed above (Supplementary Note 1), partial U reduction was observed under the beam for UO_3, even at LN2 temperatures and low-dose beam conditions. As reduction occurs rapidly in these particularly electron beam-sensitive samples, it is difficult to control and avoid during measurements. As a result, UO_3 exhibits the largest range of EELS branching ratio values of all the samples (Fig. 7) and its median value is likely an overestimate.

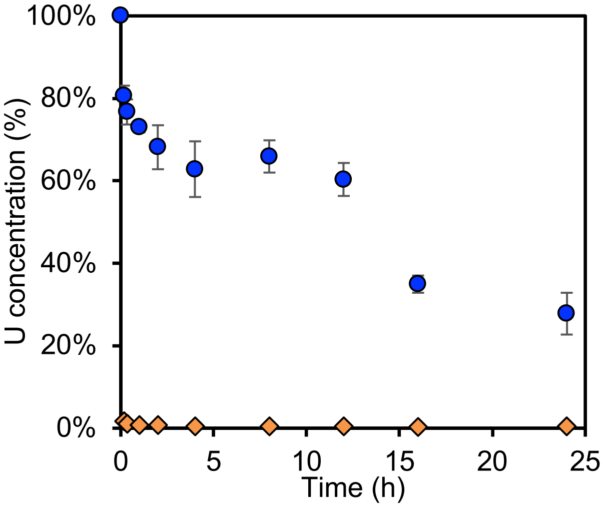

Supplementary Figure 1. Time-dependent U concentration in a magnetite-U(VI) incubation. ◇ concentration of remaining uranium in aqueous phase; ● bicarbonate extracted uranium from the magnetite suspension. The decreasing amount of bicarbonate-extractable uranium indicates that U species becomes less soluble. At 8 and 12 hours, there is a slight increase in extracted U, which might suggest the formation of intermediate U species that are extractable by bicarbonate. Replicate experiments were performed (Supplementary Table 1), and the error bars represent standard deviations (s.d.) that were calculated from at least two replicate experiment for each time point. Source data provided as a Source Data file.

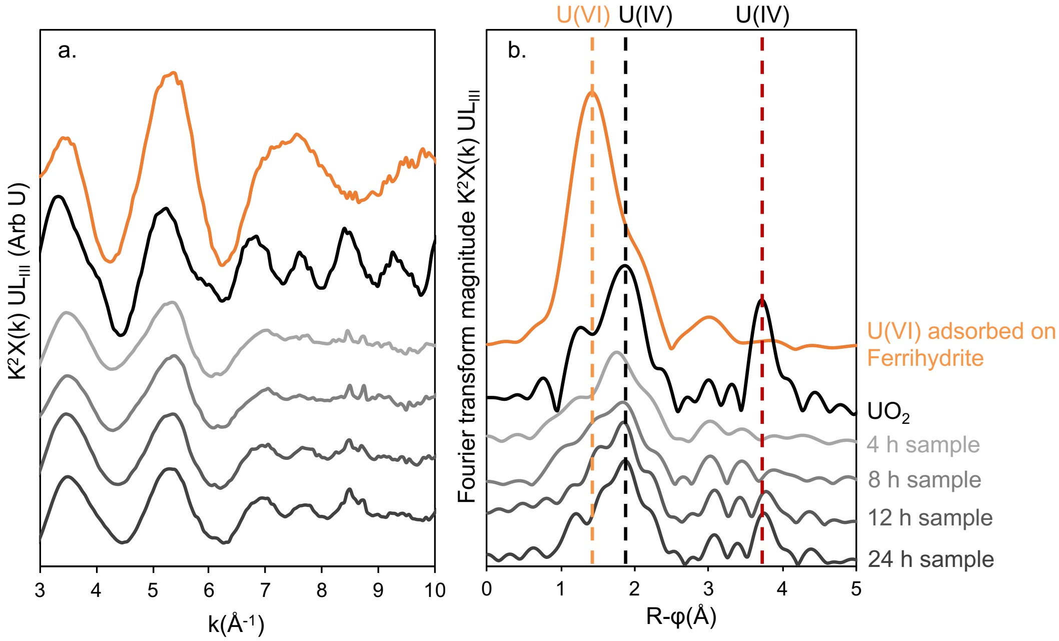

Supplementary Figure 2. X-ray absorption spectroscopy of U-magnetite solid phase. L_3 edge extended x-ray absorption fine structure (EXAFS) measurements for U(VI) reacted with magnetite after 4, 8, 12, and 24 hours. (a). k2-weighted U L_3 edge EXAFS spectra. (b). FT-EXAFS spectra in R space. Uraninite and U(VI) adsorbed on ferrihydrite[5] were used as reference standards. The Fourier-transformed EXAFS signal of samples taken in the early stages of the reduction (from 4 to 24 hours) indicates the appearance of a more crystalline structure as a function of reaction time. The 3.8 Å peak is a distinct feature for U-U pair correlation in the uraninite crystal structure, and its amplitude increases, particularly at 24 h. Sample spectra were collected at I20, DLS. U(IV) reference spectra were collected at B18, DLS. Source data provided as a Source Data file.

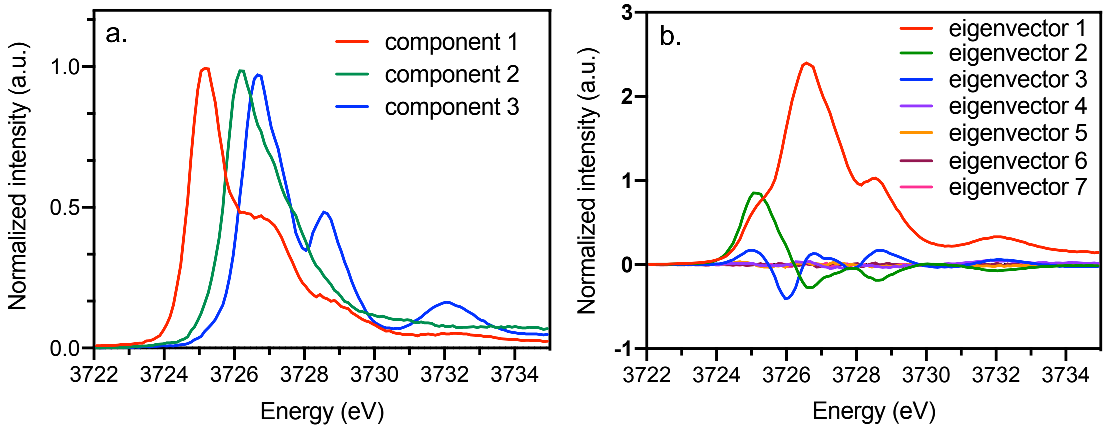

Supplementary Figure 3. Iterative-target transformation factor analysis (ITFA). M_4 edge HERFD-XANES spectral were interpreted by ITFA. (a) component spectra (the three valence state reference spectra) calculated with the ITFA program; (b) ITFA-extracted eigenvector contribution. Three eigenvectors in (b) show a signal while eigenvectors 4, 5, 6 and 7 do not contribute to the data, indicating that all spectra can be well reproduced with only three components. And the components 1, 2 and 3 in (a) identified are U(IV), U(V) and U(VI) correspondingly. Source data provided as a Source Data file.

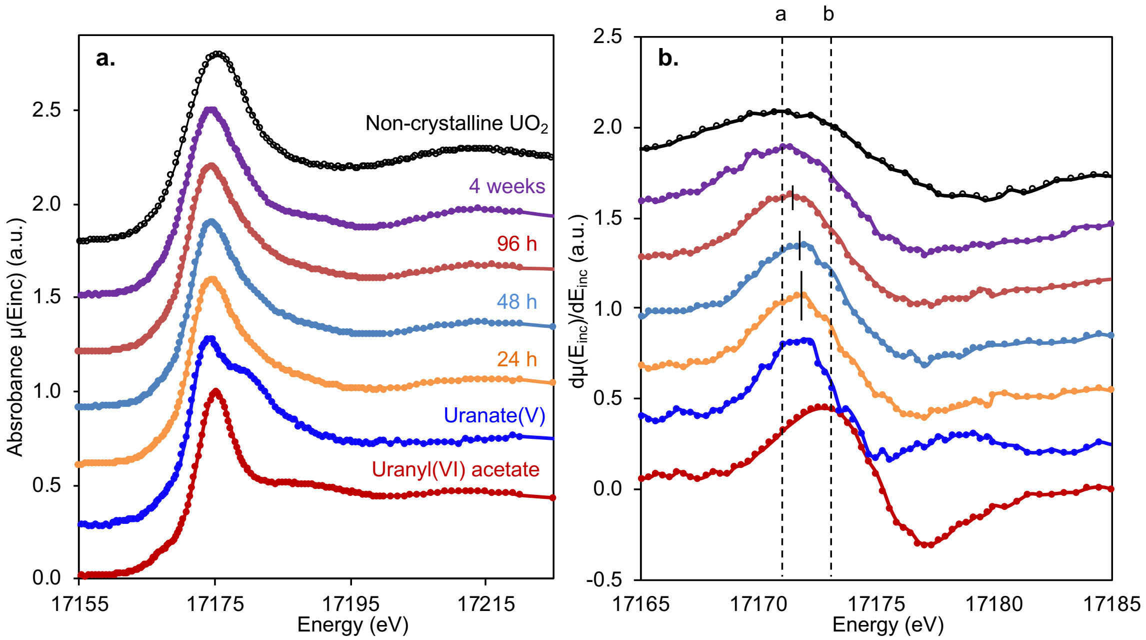

Supplementary Figure 4. X-ray absorption spectroscopy of U-magnetite solid phase. U L_3 edge HERFD-XANES spectra were obtained for U(VI) reacted with magnetite after 24, 48, 96 hours, and 4 weeks. (a) U L_3 edge HERFD-XANES spectra and (b) corresponding first derivatives for a smaller energy range compared to uranyl(VI) acetate, uranate(V) (U(V) incorporated inside magnetite structure) and noncrystalline uraninite(IV) (NCU(IV)) references. The dashed line 'a' indicates the maximum of the first derivative of NCU(IV), dashed line b shows that of U(VI). Short lines were positioned on the maximum of the first derivative for each spectrum, suggesting a decreasing trend of the inflection point from the 24-hour to the 4-week samples towards lower uranium valence states. The NCU(IV) reference spectrum (open symbols) was collected at ESRF, BM20, while all other spectra were collected at DLS, I20. Source data provided as a Source Data file.

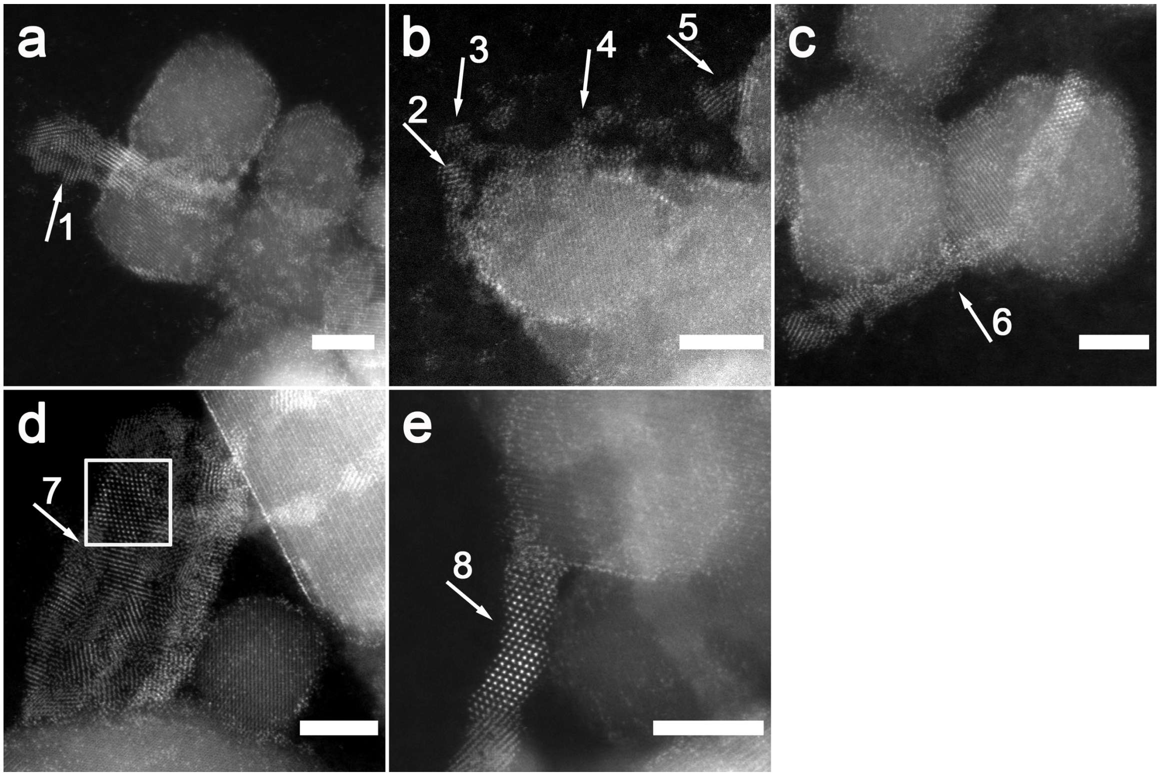

Supplementary Figure 5. Scanning transmission electron micrographs of U-magnetite samples. High-angle annular dark-field scanning transmission electron microscopy images were obtained. (a) the replicate 4-hour sample, (b) the 12-hour, (c) 24-hour, and (d and e) 72-hour samples (Scale bar = 5 nm). Arrow 1 is pointing to a short single nanowire attached to magnetite nanoparticles, representing its formation at an early time point; arrows 2-5 are pointing to individual uraninite nanoparticles on or near the surface of magnetite nanoparticles; arrow 6 is pointing to a short, single nanowire that was attached to magnetite grains; arrows 7 and 8 are pointing to uraninite nanoparticles in the nanowire structures that are ≥ 5 nm in size. The bright spots pointed to by arrows 7 and 8 are columns of U atoms in nanoparticles that appear to be well oriented to low-index crystallographic directions.

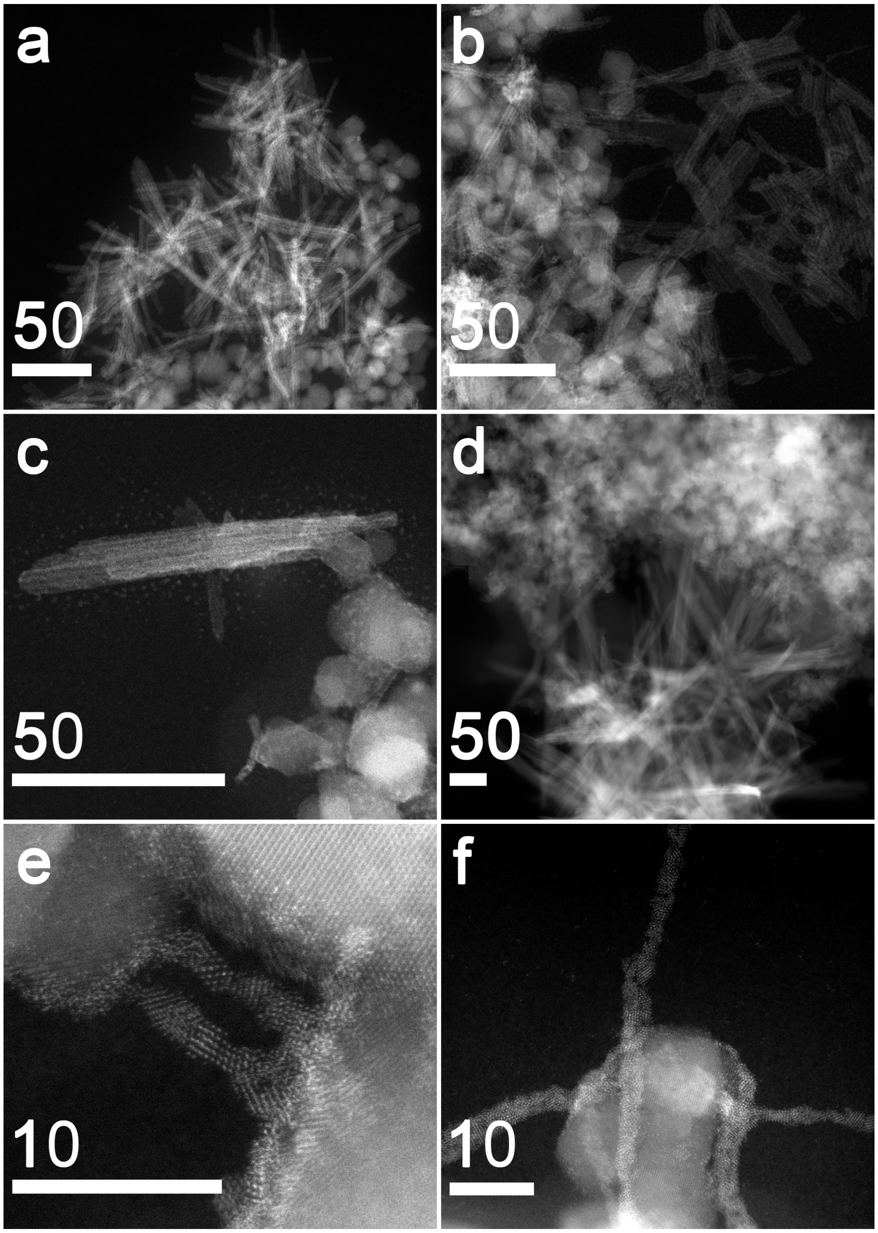

Supplementary Figure 6. Scanning transmission electron micrographs of U-magnetite samples. High-angle annular dark-field scanning transmission electron microscopy images for the 24-hour (Contr 1, 3, 4, and 5) and 72-hour (Contr 2) samples in control experiments were obtained (scale bar 50 nm or 10 nm). a. Contr 1: no magnet separation; b. Contr 2: prepared TEM grid with nanoparticles resuspended in Milli-Q water; c. Contr 3: no PIPES; d. Contr 4: second batch of magnetite stock; e. Contr 5: pH at 6.2; f. Contr 5: pH at 8. All samples show the formation of nanowires.

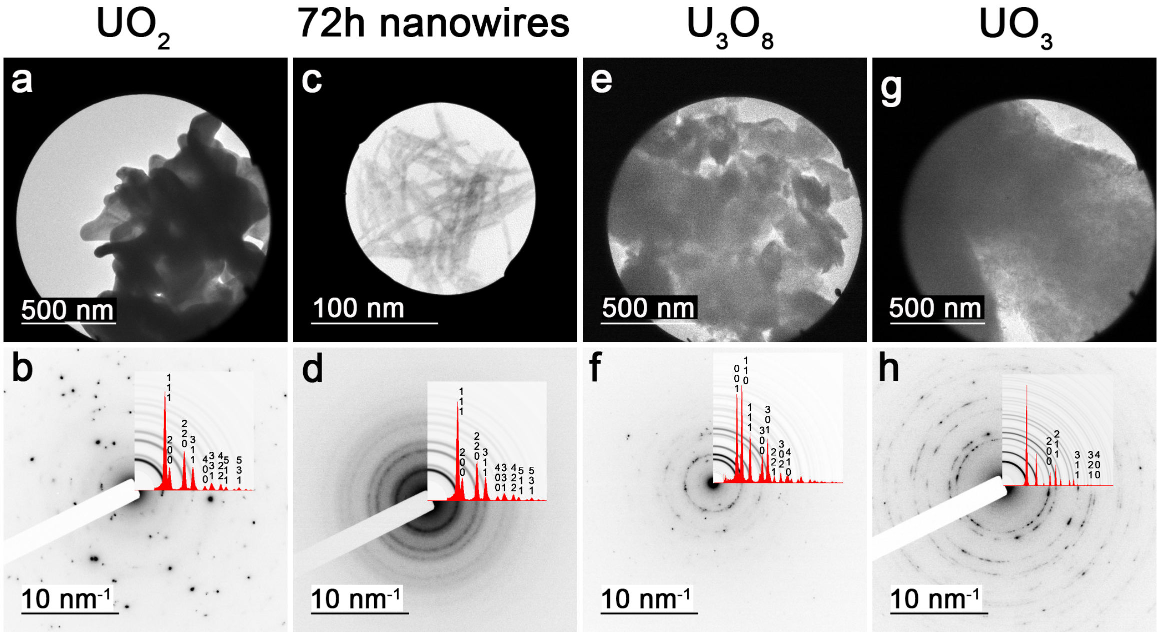

Supplementary Figure 7: Selected area electron diffraction (SAED) patterns. Bright field images of (a) UO_2 standard, (c) 72-hour nanowires structures, (e) U_3O_8 standard, and (g) UO_3 standard. SAED patterns and simulated SAED patterns with the profile for (b,d) UO_2-1541665, (f) U_3O_8-2310811, and (h) UO_3-1540845.

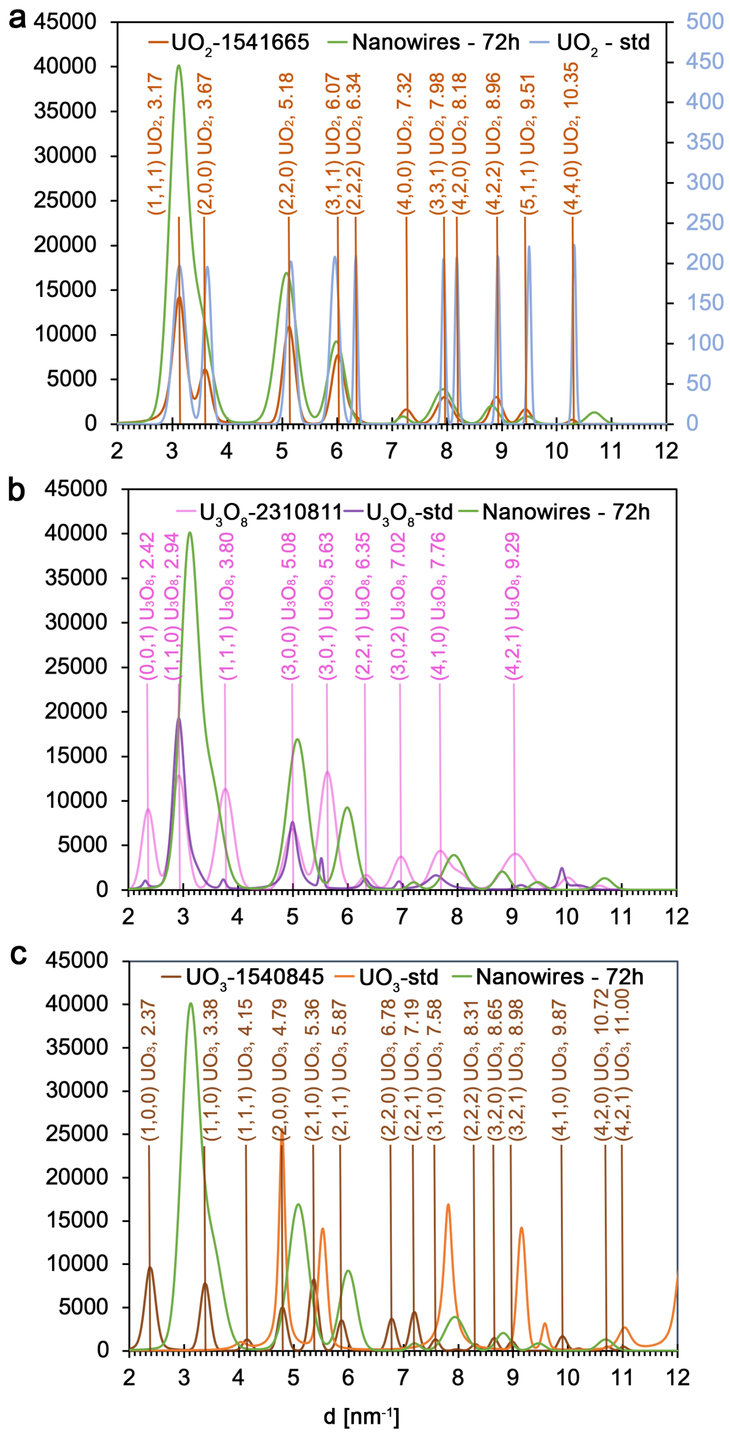

Supplementary Figure 8: Selected area electron diffraction profiles. (Scattered) profile plot of radially integrated diffraction intensity distribution with reciprocal space. (a) UO_2 standard and for UO_2-1541665, (b) U_3O_8 standard and U_3O_8-2310811 and (c) UO_3 standard and UO_3-1540845. The line plot for nanowires at 72-hour was added to each standard to show that it fits only with the UO_2 structure. Therefore, we conclude that the nanowire crystal structure is primarily the UO_2 phase and comprises uranium with a U(IV) oxidation state. Source data provided as a Source Data file.

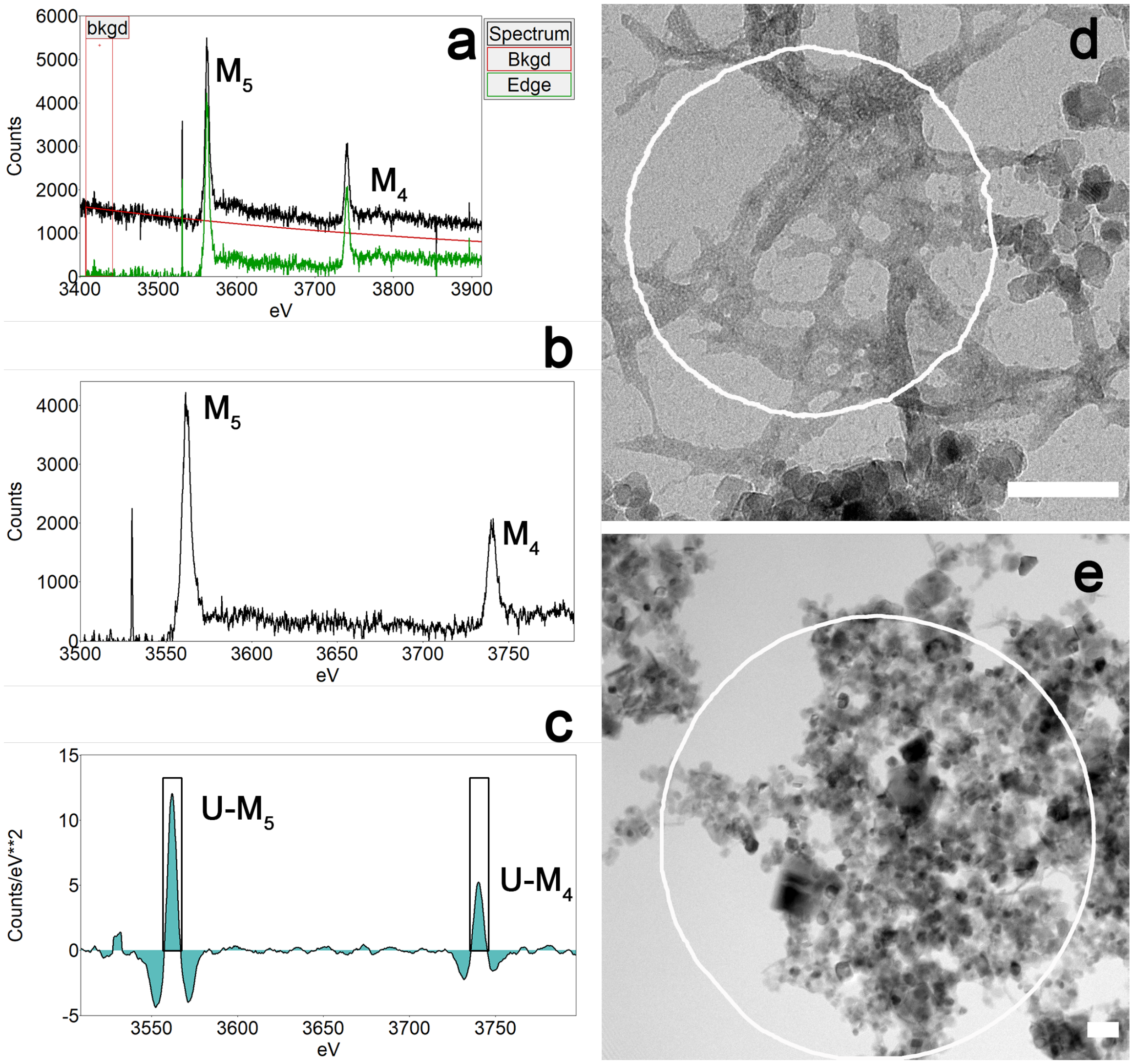

Supplementary Figure 9. Illustration of EELS spectrum analysis. (a) a background-subtracted spectrum (green) compared to the original spectrum (in black); (b) signal spectrum after background subtraction, two peaks are representative of the M_5 and M_4 edges; (c) the second derivative of the signal spectrum in (b) and two windows (in green within two boxes) over the peaks from which the integrated signal was obtained. (d) TEM images of nanowires in the 24-hour sample and (e) magnetite surface in 24-hour sample (Scale bar = 50 nm). EELS spectra were collected within the selected area (electron) diffraction aperture represented by the white circle.

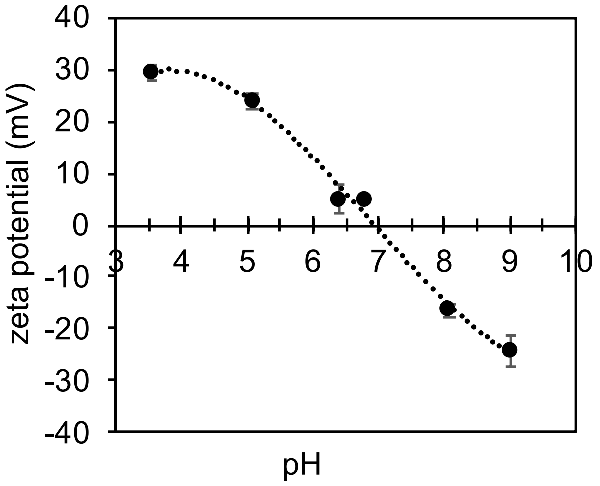

Supplementary Figure 10. Zeta potential of magnetite nanoparticles as a function of pH. Error bars represent standard deviations (s.d.) that were derived from at least 3 measurements. Source data provided as a Source Data file.

Supplementary Table 1. Experimental summary. Summary of aqueous and solid phase measurements and characterization of samples collected in replicate experiments with 200 μM U(VI), 5 mM (as Fe) magnetite, 20 mM PIPES, 1 mM $NaHCO_3$, pH 7.

Batch	ICP-MS	HAADF-STEM	XAS	EELS	magnetite
1	10-min, 20-min, 1, 2, 4, 8, 12, 24 hours	4, 12, 24 hours	-	-	Magnetite Stock 1
2	-	replicate 4-hour	-	-	
3	10-min, 20-min, 1, 2, 4, 8, 12, 24 hours	4, 12, 24, 72 hours, 24 hours (Contr 1) 72 hours (Contr 2)	M_4 edge: 4, 8, 12, 16 hours	4, 24 hours	
4	20-min, 1, 2, 4, 8, 12, 16, 24 hours		L_3 edge EXAFS: 4, 8, 12, 24 hours	-	
5	20-min, 1, 2, 4, 8, 12, 16 hours	4 weeks	L_3 edge HERFD-XANES: 24, 48, 96 hours and 4 weeks	-	
6	-	24 hours (Contr 3)	-	-	
7	-	5 days	-	-	
8	-	24 hours (Contr 4)	-	-	Magnetite Stock 2
9	-	24 hours at pH 6.2 and 8 (Contr 5)	-	-	Magnetite Stock 1

Supplementary Table 2. UO_2 crystal structure d-spacings and those obtained from the radial distribution profile for the 24-hour and 72-hour samples, as shown in Fig. 5a-d.

	d (nm^{-1})				
(h,k,l)	UO_2 (crystal structure #1541665)	Fig. 5a 24-hour	Fig. 5b 24-hour	Fig. 5c 72-hour	Fig. 5d 72-hour
(1,1,1)	3.1675	3.2435	3.2596	3.1933	3.2058
(2,0,0)	3.6575	3.7715	3.7476	3.6542	3.6764
(2,2,0)	5.1725	5.4047	5.3373	5.3616	5.2967
(3,1,1)	6.0653	6.3997	6.3568	6.2749	6.2263
(3,3,1)	7.9714	-	-	8.1768	8.1415

Supplementary Table 3. M_4, M_5-edge branching ratios obtained from EELS measurements. The median branching ratio was calculated from 10-13 measurements and the corresponding minimum, 25% percentile, 75% percentile, maximum values reported.

**Reference or Sample**	Minimum	25% Percentile	**Median**	75% Percentile	Maximum	Range	Number of measurments
UO_2	0.6898	0.6933	**0.6956**	0.6979	0.7068	0.0170	10
$UMoO_5$	0.673	0.6804	**0.6887**	0.6908	0.6927	0.0197	10
UO_3	0.6423	0.6597	**0.6640**	0.6683	0.6786	0.0363	13
24h-nanowires	0.6875	0.6943	**0.7005**	0.7079	0.7089	0.0214	11
24h-mag	0.6625	0.6718	**0.6768**	0.6855	0.6905	0.0280	13
4h-mag	0.6548	0.6666	**0.6730**	0.6775	0.6816	0.0268	13

Supplementary References

1. Stadelmann, P. A. EMS - a software package for electron diffraction analysis and HREM image simulation in materials science. *Ultramicroscopy* **21**, 131–145 (1987).
2. Gammer, C., Mangler, C., Rentenberger, C. & Karnthaler, H. P. Quantitative local profile analysis of nanomaterials by electron diffraction. *Scr. Mater.* **63**, 312–315 (2010).
3. Buck, E. C., Finn, P. A. & Bates, J. K. Electron energy-loss spectroscopy of anomalous plutonium behavior in nuclear waste materials. *Micron* **35**, 235–243 (2004).
4. Colella, M., Lumpkin, G. R., Zhang, Z., Buck, E. C. & Smith, K. L. Determination of the uranium valence state in the brannerite structure using EELS, XPS, and EDX. *Phys. Chem. Miner.* **32**, 52–64 (2005).
5. Stylo, M. *et al.* Uranium isotopes fingerprint biotic reduction. *Proc. Natl. Acad. Sci. U. S. A.* **112**, 5619–5624 (2015).
